# Use of Natriuretic Peptides in Critically Ill Patients: A Narrative Review

**DOI:** 10.3390/jcm15135244

**Published:** 2026-07-04

**Authors:** Ayodeji Olarewaju, Akinade Adebowale, Peter Odutola, Annie Arnold

**Affiliations:** 1 Department of Internal Medicine, Augusta University, Augusta, GA 30904, USA; aolarewa@phoebehealth.com; 2 Department of Psychiatry, McMaster University, Hamilton, ON L8S 4L8, Canada; adeboa1@mcmaster.ca; 3 AbbVie (United States), North Chicago, IL 60064, USA; 4 Phoebe Health, Albany, GA 31701, USA; anarnold@phoebehealth.com

**Keywords:** natriuretic peptides, critical illness, intensive care unit

## Abstract

**Background**: Natriuretic peptides, including B-type natriuretic peptide (BNP) and N-terminal pro-B-type natriuretic peptide (NT-proBNP), are established biomarkers of myocardial stress and circulatory overload. Although originally validated for diagnosis and exclusion of heart failure, their diagnostic and prognostic applications have expanded significantly in the context of critical illness. However, interpretation in critically ill patients is complicated by confounding factors such as systemic inflammation and renal dysfunction. **Objective**: This review synthesizes current evidence on the diagnostic, monitoring, and prognostic applications of natriuretic peptides in critically ill adults. It further outlines practical considerations, confounding variables, and emerging complementary biomarkers pertinent to clinical decision-making. **Methods**: A structured search of PubMed, Embase, and the Cochrane Library (January 2000 to October 2025) identified studies evaluating BNP, NT-proBNP, and atrial natriuretic peptide (ANP) in intensive care unit (ICU) patients. Eligible studies and review articles assessed diagnostic utility, volume status, hemodynamic monitoring, and prognostic performance. Narrative synthesis was employed using information obtained from eligible studies. **Results**: Twenty-four studies met the inclusion criteria. BNP and NT-proBNP facilitate differentiation between cardiogenic and noncardiogenic respiratory failure, identification of mixed shock states, and assessment of volume status when used in association with other modalities such as echocardiography and ultrasonography. Elevated natriuretic peptide concentrations consistently predict mortality, acute kidney injury, prolonged mechanical ventilation, and adverse outcomes in several disease states, including sepsis, acute respiratory distress syndrome [ARDS], postoperative cardiac dysfunction, and COVID-19-related critical illness. However, interpretation remains limited by confounders, including renal impairment, age, systemic inflammation, brain injury, mechanical ventilation, and right-ventricular strain/dysfunction. **Conclusions**: Natriuretic peptides serve as valuable adjuncts for diagnostic assessment, hemodynamic monitoring, and risk stratification in the ICU. When interpreted with attention to biological kinetics and clinical context, these biomarkers enhance multimodal monitoring and support individualized management. Future research should refine ICU-specific cutoffs and assess natriuretic peptide–guided therapeutic strategies in prospective multicenter trials.

## 1. Introduction

Critical illness is characterized by systemic inflammation, often accompanied by multisystem dysfunction and multi-organ failure [1] that carry a high burden of morbidity and mortality. The cardiovascular system is frequently affected in critically ill patients, and timely recognition of hemodynamic compromise and myocardial dysfunction is essential for guiding therapy, improving outcomes [2] and providing prognosis. Among biomarkers evaluated in the intensive care unit (ICU), B-type natriuretic peptide (BNP) and its N-terminal fragment (NT-proBNP) have emerged as valuable indicators of cardiac stress and circulatory overload [3,4]. Secreted predominantly by ventricular myocytes in response to increased wall tension, these peptides exert natriuretic, diuretic, anti-inflammatory, and vasodilatory effects while suppressing the renin–angiotensin–aldosterone and sympathetic nervous systems [5,6].

Initially validated for heart failure diagnosis and prognosis, natriuretic peptides have gained relevance across diverse critical illnesses. Elevated BNP and NT-proBNP levels occur in sepsis, acute respiratory distress syndrome, shock states, and severe trauma even without overt ventricular dysfunction [7]. Their elevation reflects ventricular wall stress, systemic inflammation, cytokine-mediated myocardial depression, and neurohormonal activation, providing integrated information linking cardiovascular mechanics to systemic inflammatory and metabolic responses [8].

There has been interest in the use of serial natriuretic peptide measurements to assist in diagnosis, risk stratification, and therapeutic decision-making in critically ill patients. Dynamic BNP changes have been investigated in the assessment of fluid balance, cardiac filling pressures, and successful ventilator liberation [9]. Elevated or rising NT-proBNP concentrations predict higher mortality and organ failure across heterogeneous ICU populations [10,11]. However, interpretation remains complex due to confounding factors including renal dysfunction, advanced age, and altered clearance. In addition, overlap between cardiac and non-cardiac causes of elevated natriuretic peptides challenges their utility in critically ill patients.

Despite these limitations, the consistent association between natriuretic peptides and adverse outcomes supports their use in the management of critically ill patients [12]. Their rapid measurement makes them attractive adjuncts to hemodynamic and echocardiographic assessment, particularly when invasive monitoring is impractical [13]. Integrating BNP-guided evaluation with clinical and physiological indices may refine fluid management, identify subclinical myocardial dysfunction, and enhance prognostication.

This narrative review synthesizes current evidence on natriuretic peptides in critically ill patients, emphasizing their diagnostic, monitoring, and prognostic implications. By exploring pathophysiologic mechanisms, interpretive challenges, and clinical applications, we delineate how BNP and NT-proBNP can serve as integral components of multimodal monitoring and management in critical illness. In addition to summarizing the available evidence, this review provides a practical framework for integrating natriuretic peptide measurements with contemporary hemodynamic, echocardiographic, and biomarker-based assessment strategies. Furthermore, it highlights key biological confounders and emerging complementary biomarkers that may enhance risk stratification and support individualized clinical decision-making in diverse ICU populations.

## 2. Biology and Kinetics of Natriuretic Peptides

### 2.1. Synthesis and Secretion

B-type natriuretic peptide (BNP) and its inactive amino-terminal fragment (NT-proBNP) are both derived from a single precursor molecule, **proBNP**, which is synthesized and stored within ventricular myocytes. Mechanical wall stretch, ischemia, or neurohormonal and cytokine stimulation trigger the cleavage of proBNP into equimolar amounts of BNP, the biologically active hormone and NT-proBNP, an inert fragment [14,15]. BNP acts via natriuretic peptide receptors A and B, generating cyclic GMP and promoting natriuresis, vasodilation, and inhibition of the renin–angiotensin–aldosterone and sympathetic nervous systems [16].

### 2.2. Clearance and Half-Life

**Figure 1:**
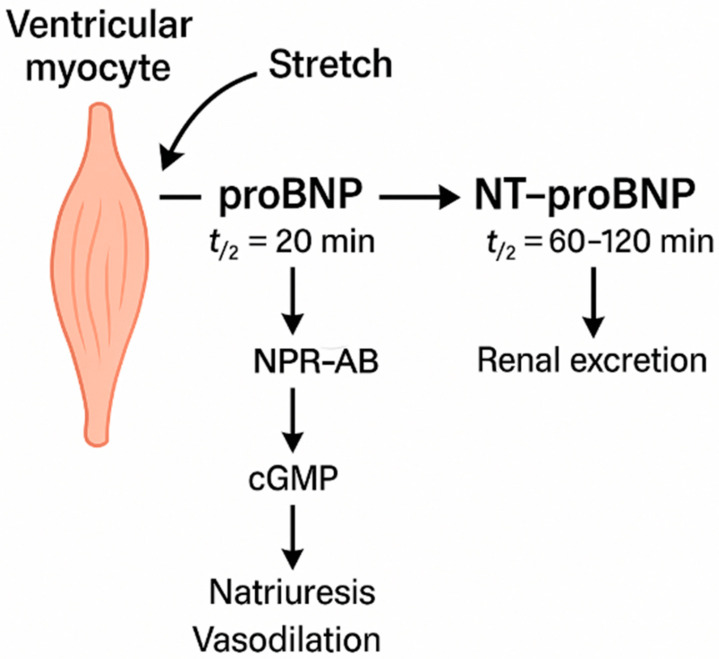
NPR-AB: Natriuretic peptide receptors A and B. cGMP: cyclic Guanosine Monophosphate. t/2: half-life.

BNP has a short plasma half-life of roughly 20 min and is removed through **receptor-mediated internalization** and degradation by **neutral endopeptidase (neprilysin)**. In contrast, NT-proBNP is biologically inactive but more stable, with a half-life of 60–120 min and is primarily cleared by the **kidneys** [17] **as shown in** Figure 1**.** Consequently, NT-proBNP levels are typically several times higher than BNP and are more significantly affected by renal dysfunction and age.

### 2.3. Regulation and Clinical Interpretation

Circulating concentrations of both peptides rise rapidly in response to ventricular volume or pressure overload. In critical illness, elevations also occur in sepsis, acute respiratory distress syndrome (ARDS), acute brain injury, pulmonary embolism, acute kidney injury and shock, reflecting an interplay of multiple processes that include myocardial strain, systemic inflammation, neurohormonal activation, impaired clearance and cytokine-mediated myocardial dysfunction, rather than isolated heart failure [14].

Understanding these biological and kinetic determinants is vital for interpreting natriuretic peptide levels in critically ill patients.

### 2.4. Emerging Biomarkers

Alongside natriuretic peptides, new markers such as **adrenomedullin, angiopoietins, renin**, and **dipeptidyl peptidase 3 (DPP3)** are being evaluated in the assessment of circulatory failure. DPP3, a cytosolic peptidase released during cellular injury, degrades vasoactive peptides including angiotensin II; increased plasma DPP3 correlates with hemodynamic dysfunction and mortality in shock [18,19].

Interpretation of these biomarkers should be placed in the context of the broader neurohormonal response to circulatory failure. Beyond activation of the renin–angiotensin–aldosterone system (RAAS), severe shock is characterized by dynamic changes in the vasopressin system. In the early phase of distributive shock, arginine vasopressin concentrations rise as a compensatory response to hypotension [20,21]. Prolonged shock, however, may result in relative vasopressin deficiency due to depletion of neurohypophyseal stores and impaired baroreceptor-mediated release. In contrast, cardiogenic shock is typically associated with sustained vasopressin activation, reflecting persistent low cardiac output and ongoing neuroendocrine stimulation [20,22]. These changes occur alongside elevations in natriuretic peptides, which indicate myocardial wall stress and circulatory overload.

Natriuretic peptides should therefore be considered components of an integrated biomarker network that captures multiple aspects of cardiovascular dysfunction, including myocardial strain, neurohormonal activation, regulation of vascular tone, and organ perfusion [23,24]. Future multimarker strategies that combine BNP or NT-proBNP with additional biomarkers such as DPP3, renin, adrenomedullin, and copeptin have the potential to enhance risk stratification and inform personalized hemodynamic management in critically ill patients [25,26,27].

## 3. Objective

To examine the evolving evidence on the use of natriuretic peptides, primarily B-type natriuretic peptide (BNP) and N-terminal pro-B-type natriuretic peptide (NT-proBNP) in critically ill adults.

## 4. Methods

### 4.1. Search Strategy

**Figure 2:**
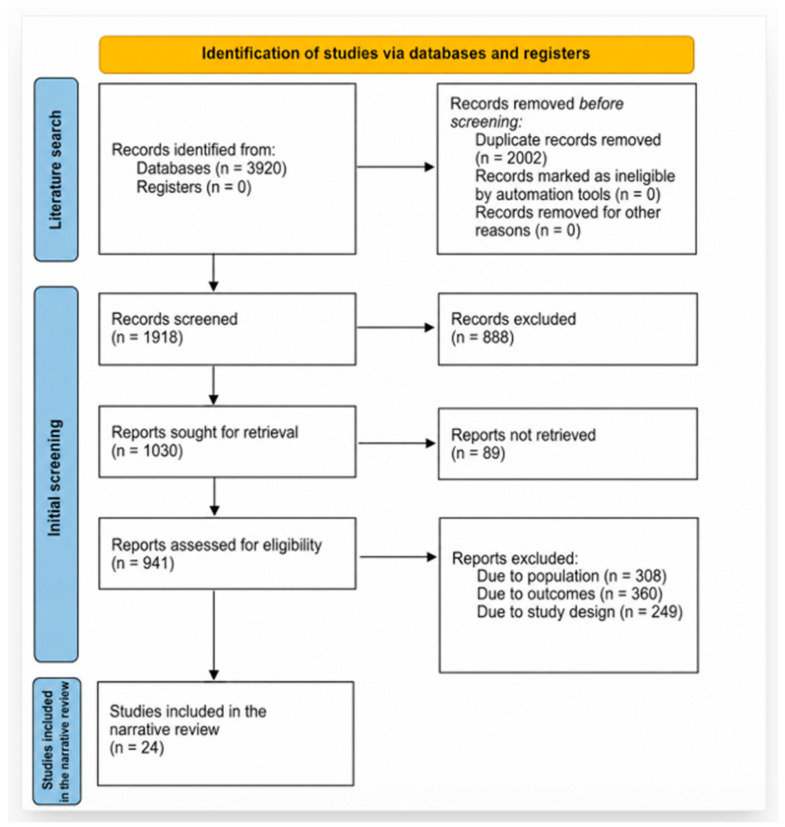
Summary of literature identification and study selection process.

A structured literature search was conducted in PubMed, Embase, and the Cochrane Library from January 2000 to October 2025 to identify relevant articles evaluating the diagnostic, prognostic, or therapeutic use of natriuretic peptides, including B-type natriuretic peptide (BNP), N-terminal pro-B-type natriuretic peptide (NT-proBNP), and atrial natriuretic peptide (ANP) in critically ill patients. The literature identification and study selection process is summarized in Figure 2. Although structured search methods, predefined eligibility criteria, and independent study screening were employed to improve transparency, this work was conducted as a narrative review and did not include protocol registration, formal risk-of-bias assessment, or quantitative evidence synthesis. Study selection was performed through title, abstract, and full-text screening based on predefined eligibility criteria.

The following keywords and MeSH terms were combined using Boolean operators: (“B-type natriuretic peptide” OR “NT-proBNP” OR “natriuretic peptides” OR “pro-BNP”) AND (“critical illness” OR “intensive care” OR “sepsis” OR “shock” OR “respiratory failure” OR “ARDS” OR “mechanical ventilation”). References for identified articles and key review papers were manually searched to identify additional eligible studies. Additional studies evaluating atrial natriuretic peptide (ANP) and mid-regional pro-atrial natriuretic peptide (MR-proANP) were identified through manual review of reference lists from eligible studies and relevant review articles.

### 4.2. Eligibility Criteria

Studies were included if they:

1. Enrolled critically ill patients (medical, surgical, or mixed ICU).
2. Reported BNP or NT-proBNP levels measured for diagnostic, monitoring, or prognostic purposes.
3. Evaluated clinical outcomes such as cardiac dysfunction, hemodynamic parameters, ventilator weaning, acute kidney injury, or mortality.
4. Were original investigations (randomized controlled trials, cohort studies, cross-sectional studies) or systematic/narrative reviews with ICU focus.

Studies were excluded if they:

1. Included only outpatients or non-critical chronic populations (e.g., stable heart failure or pulmonary hypertension).
2. Were animal studies, case reports, conference abstracts.
3. Non-English language publications.

### 4.3. Study Selection and Data Extraction

Two reviewers independently screened titles and abstracts for eligibility, followed by full-text review. For each included study, the following data were extracted:

1. Study design and population (ICU setting, diagnosis, sample size);
2. Type of natriuretic peptide measured (BNP, NT-proBNP, ANP, MR-proANP);
3. Primary objective (diagnostic, monitoring, or prognostic);
4. Main outcomes and key findings;
5. Limitations and interpretive considerations.

### 4.4. Data Were Organized into Two Evidence Tables

Table 1 (part A): Diagnostic and prognostic studies. Table 1 (part B): Therapeutic and mechanistic studies.

### 4.5. Data Synthesis and Thematic Categorization

Given the heterogeneity of patient populations, assay methods, and clinical outcomes, quantitative pooling was not performed. Instead, results were synthesized narratively and grouped into three primary themes:

1. Diagnostic utility—distinguishing cardiogenic vs. noncardiogenic shock or pulmonary edema.
2. Monitoring role—tracking hemodynamic stress, volume status, and therapy response.
3. Prognostic value—association with mortality, ICU stay, and organ failure.

Special attention was paid to confounding variables affecting peptide levels, including renal dysfunction, sepsis-induced myocardial depression, systemic inflammation, right ventricular strain, and mechanical ventilation.

### 4.6. Quality Assessment

Although formal bias scoring was not conducted (given the narrative scope), study design, sample size, assay standardization, and adjustment for confounders were qualitatively appraised to gauge evidence robustness. Higher-weight consideration was given to prospective trials, systematic reviews, and meta-analyses, while older or single-center studies were used for contextual support.

## 5. Results

A total of 24 studies met the inclusion criteria and were included in this review. Of these, 16 investigations focused primarily on the diagnostic and prognostic utility of natriuretic peptides in critically ill adults, while 8 studies explored their therapeutic and mechanistic implications. Together, the studies encompassed heterogeneous ICU populations, including patients with sepsis, shock, acute respiratory distress syndrome (ARDS), pulmonary embolism, systemic inflammatory response syndrome, postoperative cardiac dysfunction, and COVID-19–related critical illness.

Most studies evaluated B-type natriuretic peptide (BNP) and/or its inactive fragment N-terminal pro-B-type natriuretic peptide (NT-proBNP), while a few assessed atrial natriuretic peptides (ANP). Study designs ranged from randomized controlled trials and prospective cohorts to systematic reviews, meta-analyses, and narrative syntheses. Sample sizes varied widely, reflecting the evolution of evidence from early single-center investigations to multicenter studies and meta-analysis.

A detailed summary of diagnostic and prognostic evidence is presented in Table 1 (part A), while therapeutic and mechanistic insights are summarized in Table 1 (part B). Table 2 provides an overview of the kinetics and key determinants of BNP and NT-proBNP in critical illness, emphasizing how biological differences and ICU-specific confounders influence peptide interpretation.

Table 2 summarizes the principal diagnostic, prognostic, and monitoring applications of BNP and NT-proBNP across common ICU conditions. Biomarker interpretation should be integrated with clinical assessment, imaging findings, and underlying comorbidities.

## 6. Discussion

Natriuretic peptides have emerged as essential adjuncts in the evaluation of critically ill patients, particularly those with acute cardiopulmonary decompensation. In the intensive care unit (ICU), accurate interpretation of BNP and NT-proBNP levels requires integration with other biomarkers as well as hemodynamic and echocardiographic findings. Several investigations have demonstrated that both peptides show a weak correlation with pulmonary capillary wedge pressure and left-ventricular filling pressures, and in certain patients, they can be employed as part of a multimodal approach to distinguish cardiogenic from non-cardiogenic pulmonary edema. However, diagnostic specificity diminishes in the ICU due to confounding factors such as renal impairment, mechanical ventilation, medications, and systemic inflammation, which independently alter natriuretic peptide levels. In addition, traditional emergency-department cutoffs lose reliability in older or ventilated patients.

Importantly, there are currently no universally accepted ICU-specific diagnostic thresholds for BNP or NT-proBNP. Although BNP concentrations <100 pg/mL have traditionally been used to exclude heart failure in emergency department populations, studies conducted in critically ill patients have reported substantially different cutoff values. For example, Tung et al. identified a BNP threshold of 350 pg/mL for differentiating cardiogenic from non-cardiogenic pulmonary edema in mechanically ventilated patients. These discrepancies likely reflect the influence of factors commonly encountered in the ICU, including advanced age, renal dysfunction, systemic inflammation, mechanical ventilation, and right ventricular strain. Consequently, natriuretic peptide concentrations should not be interpreted using a single universal threshold in critically ill patients. Rather, BNP and NT-proBNP values should be integrated with clinical assessment, echocardiography, lung ultrasonography, and other hemodynamic parameters to support diagnostic and therapeutic decision-making.

**Figure 3:**
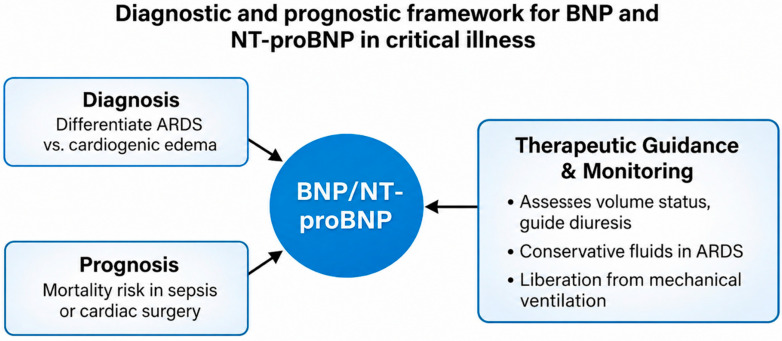
Mechanisms contributing to BNP and NT-proBNP elevation in critically ill patients. Natriuretic peptide concentrations may increase due to ventricular wall stress, myocardial dysfunction, pulmonary vascular disease, renal impairment, systemic inflammation, sepsis-associated myocardial injury, and volume overload. Multiple mechanisms frequently coexist in ICU patients [28,29,30,31,32,33,34,36].

In septic or postoperative shock, myocardial depression and cytokine-mediated dysfunction frequently elevate BNP level independent of preload, but extremely high levels (>5000 pg/mL) coupled with low ejection fraction or elevated filling pressures often reliably indicate a cardiogenic component. The diagnostic and prognostic framework in critical illness is represented in Figure 3.

Beyond diagnostic use, natriuretic peptides serve as surrogate markers of myocardial stretch and congestion rather than absolute volume status. Contemporary critical care practice prioritizes multimodal hemodynamic assessment over reliance on a single biomarker or monitoring technique [51]. Natriuretic peptides yield the greatest clinical value when interpreted in conjunction with advanced bedside echocardiographic and ultrasonographic parameters [52,53]. For instance, elevated BNP or NT-proBNP concentrations combined with an increased E/e′ ratio can provide stronger evidence of elevated left ventricular filling pressures [54,55]. Concurrent elevation of pulmonary artery systolic pressure (PASP) may indicate pulmonary vascular congestion or right ventricular strain [56,57]. Additionally, assessment of left ventricular outflow tract velocity–time integral (LVOT VTI) offers insight into stroke volume and cardiac output, facilitating differentiation of whether elevated natriuretic peptide concentrations are attributable to myocardial dysfunction, volume overload, or systemic inflammatory stress [58].

Natriuretic peptide measurements should also be integrated with dynamic indices of fluid responsiveness, such as passive leg raising-induced changes in stroke volume, pulse pressure variation, stroke volume variation, and inferior vena cava ultrasonography [59]. In this context, elevated BNP or NT-proBNP levels may reflect increased myocardial wall stress or venous congestion, while dynamic parameters help determine whether further fluid administration is likely to enhance cardiac output [60]. Combining biomarker data with functional hemodynamic assessment may improve diagnostic accuracy and facilitate more individualized fluid and vasoactive management strategies in critically ill patients. BNP has been shown to have a weak correlation with invasive measures such as wedge pressure and ventricular filling [45,61], while NT-proBNP levels are increasing with positive cumulative fluid balance and the development of ARDS [50]. Serial measurements may offer additional insight: declining levels during ventilator weaning has potential to predict extubation [38], whereas persistently high or rising concentrations predict adverse outcomes [42]. However, these dynamic changes must be interpreted in conjunction with renal function, use of vasoactive medications, and ventilator settings. Evidence remains mixed regarding BNP’s ability to reduce invasive monitoring, and it cannot replace pulmonary-artery catheterization in heterogeneous ICU shock populations [28,29].

The prognostic significance of natriuretic peptides extends across multiple ICU populations. In sepsis and septic shock, elevated BNP and NT-proBNP levels consistently predict mortality and organ dysfunction [37,42]. Similar findings are reported in ARDS, where increased concentrations predict longer mechanical ventilation and worse oxygenation [48,50]. Post-cardiac surgery, high postoperative BNP values correlate with prolonged ICU stay and mortality [43]. Likewise, BNP elevation predicts acute kidney injury and renal replacement therapy, likely reflecting venous congestion and renal venous hypertension [31,34]. During viral critical illnesses such as COVID-19, meta-analyses confirm that BNP and NT-proBNP predict both disease severity and mortality [39,40], underscoring their role as global indicators of cardiovascular stress rather than isolated cardiomyocyte dysfunction. Absolute cutoffs for predicting outcomes in critically ill patients need to be established.

Therapeutically, BNP-guided strategies have shown promise but remain unproven in general ICU populations. In a randomized controlled trial, BNP-guided fluid management shortened mechanical ventilation duration and optimized fluid balance in patients with left ventricular systolic dysfunction [49]. Studies in ARDS have demonstrated that elevated BNP during ventilator weaning predicts extubation failure and supports conservative fluid strategies [38,50]. Nevertheless, clinicians are advised to interpret BNP trends as indicators of persistent myocardial strain rather than absolute therapeutic targets. Rising levels despite diuresis should prompt reassessment for unresolved ventricular dysfunction or right-heart strain [45,48]. There is no robust evidence to support the routine use of serial BNP or NT-proBNP levels to assess volume status in critically ill patients. Therefore, in critically ill patients, BNP and pro-BNP levels should be viewed as decision-support biomarkers rather than a standalone guide for fluid therapy.

Multiple physiologic and pathologic confounders further complicate interpretation of natriuretic peptide levels in critical illness. Renal dysfunction elevates NT-proBNP even in the absence of volume overload [17], while advanced age and chronic left-ventricular hypertrophy produce higher baseline concentrations [14,16]. Sepsis-induced cardiomyopathy, mechanical ventilation with high positive end-expiratory pressure, and right-ventricular strain may also spuriously elevate BNP [44,48]. Conversely, obesity can result in deceptively low values due to enhanced peptide clearance [14,16]. BNP levels are falsely elevated by Neprilysin inhibitor therapy and taken together these findings highlight that while natriuretic peptides are powerful markers of myocardial stress, their diagnostic and prognostic accuracy depends heavily on context, comorbidities, and concurrent physiologic variables.

Another important consideration in critically ill patients is right ventricular dysfunction. Although BNP is traditionally associated with left ventricular dysfunction, right ventricular pressure and volume overload can also stimulate natriuretic peptide release. Elevated BNP and NT-proBNP concentrations have been observed in pulmonary hypertension, acute pulmonary embolism, ARDS-associated pulmonary vascular dysfunction, and right ventricular failure [62,63]. In critically ill patients, elevated BNP levels should therefore be interpreted within the broader hemodynamic context, as isolated increases do not necessarily indicate left-sided heart failure. Integration with echocardiographic assessment of right ventricular size, function, tricuspid regurgitation velocity, and pulmonary artery systolic pressure is recommended [63].

Common misconceptions on the use of BNP and NT-proBNP in the ICU include elevated BNP equals heart failure, daily serial BNP levels can be used to track volume status and guide diuretic therapy, and the same diagnostic cutoffs apply in critically ill patients for the diagnosis of heart failure.

## 7. Emerging Biomarkers in Critical Illness

I. Soluble ST2 (sST2): Soluble ST2 is a marker of myocardial stress and fibrosis that has demonstrated prognostic utility in heart failure and critical illness. Unlike BNP, sST2 appears less influenced by age, obesity, or renal dysfunction [64].
II. Galectin-3: Galectin-3 reflects inflammation and myocardial fibrosis and has been associated with adverse cardiovascular outcomes. Combined assessment of BNP and Galectin-3 may improve risk stratification [65].
III. High-Sensitivity Troponin (hs-cTn): High-sensitivity cardiac troponin frequently rises in critically ill patients and may indicate myocardial injury related to sepsis, ischemia, or ventricular strain. Concurrent elevations of BNP and hs-cTn are often associated with worse outcomes [66].
IV. CT-1 (Cardiotrophin-1): Cardiotrophin-1 is a cytokine involved in myocardial hypertrophy and ventricular remodeling. Emerging evidence suggests potential prognostic utility in cardiovascular disease, although ICU-specific data remain limited [67].
V. MR-proADM: Mid-regional pro-adrenomedullin (MR-proADM) has emerged as a marker of endothelial dysfunction and disease severity in sepsis and critical illness [68].

## 8. Practical Bedside Framework/Proposed Algorithm

Integrating natriuretic peptides with echocardiography and ultrasound enhances bedside decision-making and improves care of critically ill patients. Hypoxemia with bilateral infiltrates: Low natriuretic peptide concentrations may reduce the likelihood of cardiogenic pulmonary edema, whereas markedly elevated levels should prompt further evaluation with echocardiography and comprehensive hemodynamic assessment. Given the influence of age, renal dysfunction, systemic inflammation, mechanical ventilation, and right ventricular strain, natriuretic peptide concentrations should not be interpreted using a single universal cutoff in critically ill patients [28,29,30]; Oliguric AKI with fluid overload: Rising BNP with a plethoric inferior vena cava and pulmonary venous congestion on ultrasound supports decongestion therapy, whereas falling BNP with a collapsible IVC suggests intravascular depletion [31,34,38,42,50]; Early sepsis: Rapid BNP elevation signals cardiovascular stress; intensified monitoring and echocardiographic assessment are recommended [29,38,42]. Increasingly, natriuretic peptide interpretation is integrated with point-of-care ultrasound (POCUS) in critically ill patients. BNP and NT-proBNP provide biochemical evidence of myocardial wall stress, whereas focused cardiac ultrasound, lung ultrasound, and venous congestion assessment provide complementary hemodynamic information [53]. Combined use of natriuretic peptides and bedside ultrasound may improve diagnostic accuracy, particularly in differentiating cardiogenic from non-cardiogenic causes of respiratory failure and guiding fluid management decisions [69].

**Figure 4:**
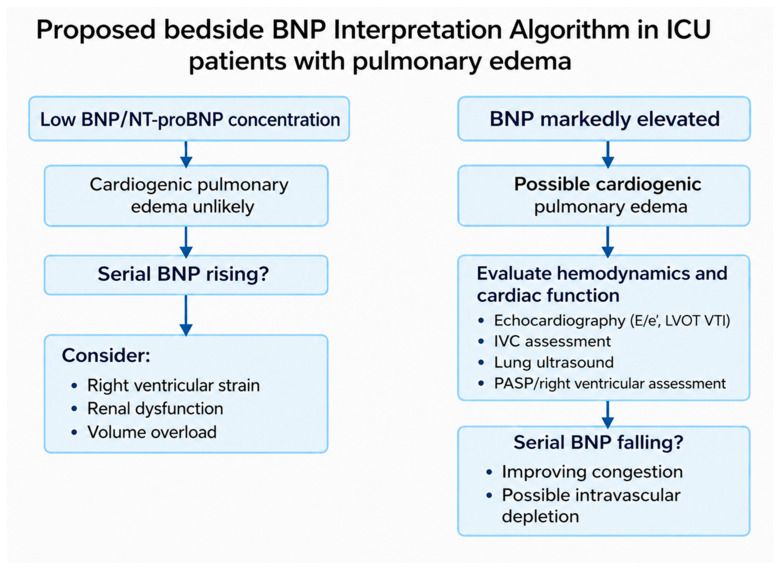
Proposed bedside interpretation algorithm for BNP and NT-proBNP in ICU patients with pulmonary edema. Low natriuretic peptide concentrations reduce the likelihood of cardiogenic pulmonary edema, whereas markedly elevated levels support further hemodynamic assessment. Interpretation should incorporate echocardiography, IVC evaluation, lung ultrasound, right ventricular assessment, and serial biomarker trends because BNP concentrations may also be influenced by renal dysfunction, pulmonary hypertension, and systemic illness [28,29,30,34,38,42,48,50].

This framework (Figure 4) emphasizes that BNP guides reassessment rather than dictates therapy.

## 9. Gaps in Evidence and Future Directions

Despite available data, key uncertainties remain. No validated ICU-specific cutoffs adjust for renal function, ventilation mode, or obesity [17]. Randomized trials are needed to determine whether BNP-guided de-resuscitation improves ventilator-free days or renal recovery [49]. Emerging multi-marker strategies integrating BNP with adrenomedullin, renin, and dipeptidyl peptidase 3 (DPP3) may better characterize circulatory stress [18,43]. Future research should also explore right-heart-focused management in ARDS and pulmonary embolism, where BNP elevation often reflects right ventricular (RV) rather than left ventricular (LV) dysfunction [48].

## 10. Study Limitations

The findings of this review should be interpreted considering several limitations. The included studies were heterogeneous in terms of patient populations, methodologies, and outcome definitions, which may have affected the comparability of BNP and NT-proBNP results across settings. Additionally, much of the existing evidence is derived from single-center or observational cohorts, which limits generalizability. These factors highlight the need for large, prospective, multicenter studies to validate natriuretic peptide-guided strategies and establish standardized interpretation frameworks for critically ill patients. Several included references consisted of narrative reviews, consensus statements, and expert opinion articles, reflecting the evolving nature of this field. Consequently, some conclusions are based on synthesized evidence rather than exclusively on primary investigations.

## 11. Conclusions

Natriuretic peptides offer valuable, non-invasive insight into cardiovascular stress and volume status in critically ill patients. Elevated BNP and NT-proBNP levels reflect myocardial wall tension driven by preload, afterload, inflammation, and neurohormonal activation—making them powerful integrative markers rather than disease-specific tests. When used in combination with other markers in the ICU, they can support the differentiation between cardiogenic and noncardiogenic pulmonary edema, guide fluid resuscitation strategies, and provide robust prognostic information in various diseases.

However, their interpretation is highly context dependent. Renal dysfunction, mechanical ventilation, and right-ventricular strain can markedly alter levels, underscoring the need to pair peptide data with imaging, other biomarkers, and clinical assessment. A low BNP remains a strong rule-out tool for cardiogenic edema, while dynamic trends—rather than absolute values—may best reflect clinical trajectory.

Future work should aim to define ICU-specific thresholds, validate BNP-guided fluid strategies, and integrate multi-marker panels that capture both left- and right-sided cardiac stress. Used thoughtfully, natriuretic peptides can enhance precision monitoring and improve hemodynamic management in critical care.

## Figures and Tables

**Table 1:** (**A**) Diagnostic and prognostic studies of natriuretic peptides in critically ill patients. (**B**) Intervention, prognostic and mechanistic studies of natriuretic peptides in critically ill patients.

(A)								
First Author (Year)	Study Type/Design	Population and Setting	Natriuretic Peptide(s) Assessed	Clinical Context/Comparator	Outcomes Assessed	Main Findings (Summary)	Interpretation Category	Comments/Limitations
Tung et al. (2004) [28]	Clinical investigation	Adult ICU; shock with PA catheter	BNP	Hemodynamics (PCWP, CI) across shock phenotypes	Diagnostic discrimination; mortality	BNP concentrations did not correlate with interpatient cardiac index or pulmonary artery occlusion pressure. BNP < 350 pg/mL had a negative predictive value of 95% for the diagnosis of cardiogenic shock Higher BNP predicts mortality.	Diagnostic + Prognostic	Heterogeneous shock etiologies; single center.
Januzzi et al. (2006) [29]	Prospective cohort	Adult ICU; mixed shock states	NT-proBNP	Pulmonary capillary wedge pressure	Diagnostic accuracy; ICU mortality	Elevated levels of NT-proBNP do not necessarily correlate with high filling pressures among patients with ICU shock, but marked elevation in NT-proBNP is strongly associated with ICU death.	Diagnostic + Prognostic	Single center; modest sample size.
Sturgess et al. (2006) [30]	Narrative review	Critically ill adults; heterogeneous ICU conditions	BNP	—	Cardiac dysfunction; disease severity; mortality	Synthesized early evidence linking BNP to myocardial dysfunction and worse outcomes.	Diagnostic + Prognostic	Older evidence base; no primary data.
McLean et al. (2008) [31]	Bench-to-bedside review	ICU; cardiac and septic conditions	BNP; NT-proBNP	—	Diagnostic and prognostic utility	Summarizes integration with troponin and echo for ICU decision-making.	Diagnostic + Prognostic	Narrative; foundational but older.
Mueller et al. (2008) [32]	Clinical review	ICU (HF, sepsis, shock)	BNP; NT-proBNP	—	Diagnostic use; volume status; outcomes	Outlined ICU applications and pitfalls; supported adjunctive rather than stand-alone use, with the most promising indications being the diagnosis of hypoxic respiratory failure and timing of extubation.	Diagnostic + Prognostic	Review; not systematic.
Pirracchio et al. (2008) [33]	Narrative review	Adult ICU; mixed conditions	BNP; NT-proBNP	—	Differentiation of shock; weaning; sepsis outcomes	BNP supports differentiation of cardiogenic vs. noncardiogenic causes and prognostication.	Diagnostic + Prognostic	Early era assays; evolving cutoffs.
Noveanu et al. (2009) [34]	Narrative review	Adult ICU; cardiac and noncardiac critical illness	BNP; NT-proBNP	Troponin level and hemodynamics	Diagnostic performance; mortality	BNP has excellent prognostic value in ICU patients, but diagnostic properties remain ambiguous and require further evaluation.	Diagnostic + Prognostic	Narrative synthesis; limited quantitative pooling.
Ristagno et al. (2010) [35]	Review	Post–cardiac arrest ICU	BNP; NT-proBNP	Troponins	Survival; Myocardial injury detection; defibrillation prediction	Higher natriuretic peptide levels linked to worse post-arrest outcomes.	Prognostic	Review-level evidence; heterogeneity across cohorts.
Luchner et al. (2012) [36]	Prospective randomized multicenter	ED/ICU	NT-proBNP	Compared with standard assessment	Hospitalization; ICU admission; mortality	NT-proBNP improved stratification of patient care and is strongly correlated with subsequent hospital resource utilization and prognosis; some ICU relevance.	Diagnostic + Prognostic	ED-based; indirect ICU applicability.
Suzuki et al. (2016) [37]	Narrative review	ED/ICU	BNP; NT-proBNP	—	HF diagnosis	Both BNP and NT-proBNP are elevated in pulmonary diseases with hemodynamic stress	Prognostic	Sample size moderate; confounding by renal dysfunction.
Deschamps et al. (2020) [38]	Systematic review and meta-analysis	Mechanically Ventilated Adults in ICU	BNP	—	Weaning success/failure; extubation outcomes	The relative change in BNP during a SBT has potential value as an incremental tool after successful SBT to predict successful liberation from MV in adults.	Diagnostic + Prognostic	Heterogeneous cutoffs; variable assay platforms.
Walker et al. (2020) [39]	Meta-analysis	Severe/critical COVID-19	BNP; NT-proBNP	—	Disease severity; mortality	Elevated cardiac biomarkers including natriuretic peptide level associated with severe disease and death.	Prognostic	Primarily COVID cohorts; variable definitions of severity.
Zhu et al. (2021) [40]	Systematic review and meta-analysis	Severe/critical COVID-19	NT-proBNP	—	Acute cardiac injury; disease severity; mortality	NT-proBNP elevation correlated with critical illness and non-survival.	Prognostic	High heterogeneity; pandemic-era data with quality variation.
Lee et al. (2023) [41]	Narrative review	General adult heart failure population	BNP, NT-proBNP	—	Mortality; cardiac dysfunction	Natriuretic peptides are well established for diagnosis of heart failure in patients presenting to the emergency department with dyspnea. Higher natriuretic peptide levels equal greater risk of adverse outcomes	Diagnostic and Prognostic	Does not address ICU applicability
Naidoo et al. (2023) [42]	Retrospective cohort	Mixed adult ICU (medical/surgical/trauma)	BNP	—	ICU mortality	BNP ≥ 366 ng/L independently predicted ICU mortality; stronger in non-HF patients.	Prognostic	Single center; retrospective design.
Jozwiak et al. (2024) [43]	Narrative review	ICU; cardiogenic/mixed shock	BNP; NT-proBNP	Multi-marker context	Mortality; shock severity	Positions BNP as part of a multi-marker strategy in shock.	Prognostic	Review; proposes integrated algorithms.
(**B**)								
**First Author (Year)**	**Study Type/Design**	**Population and Setting**	**Natriuretic Peptide(s) Assessed**	**Clinical Context/Comparator**	**Outcomes Assessed**	**Main Findings (Summary)**	**Interpretation Category**	**Comments/Limitations**
Witthaut et al. (2004) [44]	Mechanistic review	Critical illness (sepsis, trauma, surgery)	ANP; BNP; NT-proBNP	Cytokine/endotoxin pathways	Pathophysiology (myocardial dysfunction, vascular tone)	Inflammatory mediators can upregulate BNP independent of wall stress.	Mechanistic	Review; hypothesis-generating.
Forfia et al. (2005) [45]	Prospective clinical study	Adult ICU; shock/HF needing invasive hemodynamic monitoring	BNP, NT-proBNP	PCWP; invasive hemodynamics	Filling pressures; outcomes	BNP shows weak correlation to wedge pressure in critically ill.	Mechanistic + Diagnostic	Sample size/center limitations.
Meisner et al. (2005) [46]	Review/observational (contextual)	ICU; sepsis and inflammation	ANP, BNP	PCT, CRP	Sepsis severity; outcomes	Highlights interplay of cardiac stress and inflammation affecting biomarkers.	Mechanistic	Mixed designs; contextual synthesis.
Thygesen et al. (2011) [47]	Consensus guideline	General Cardiology (applies to ICU)	BNP, NT-proBNP	—	Classification framework	Provides standardized context for myocardial injury definitions used in ICU studies.	Diagnostic	Not ICU-specific; supportive reference.
Ventetuolo et al. (2011) [48]	Review	ICU; ARDS and acute RV failure	BNP; NT-proBNP	Echo markers of RV strain	RV dysfunction; outcomes	Elevated BNP level reflects increased RV load and correlates with outcomes in ARDS/PE physiology.	Mechanistic + Prognostic	Limited interventional data.
Dessap et al. (2012) [49]	Randomized controlled trial	Adult ICU; ventilator weaning phase	BNP	BNP-guided fluid management vs. usual care	Time to weaning; extubation success	BNP-driven strategy increased the number of ventilator-free days but did not change length of stay or mortality in patients with LV dysfunction.	Intervention	Need for external validation; protocol adherence considerations.
Determann et al. (2013) [50]	Secondary analysis of RCT	Mechanically Ventilated Adult ICU	NT-proBNP	Different tidal volumes; ARDS development	Fluid balance; ARDS; AKI	No correlation between NT-pro BNP level and tidal volume but higher levels observed in ARDS, AKI, and in patients with a more positive cumulative fluid balance.	Mechanistic	Exploratory; not powered for clinical endpoints.
Pickkers et al. (2021) [18]	Review	ICU; circulatory shock	NT-proBNP (+ novel biomarkers)	Adrenomedullin, renin, DPP3	Shock severity; mortality	Supports NT-proBNP as a useful prognostic biomarker in circulatory shock.	Mechanistic + Prognostic	Narrative; calls for multimodal algorithms.

AKI—Acute Kidney Injury; ANP—Atrial Natriuretic Peptide; ARDS—Acute Respiratory Distress Syndrome; BNP—B-type Natriuretic Peptide; CI—Cardiac Index; CRP—C Reactive Protein; DPP3—Dipeptidyl Peptidase 3; ED—Emergency Department; HF—Heart Failure; ICU—Intensive Care Unit; NT-proBNP—N-terminal pro–B-type Natriuretic Peptide; PA—Pulmonary Artery; PCWP—Pulmonary Capillary Wedge Pressure; PCT—Procalcitonin; PE—Pulmonary Embolism; RV—Right Ventricle.

**Table 2:** Kinetic characteristics and major confounders of BNP and NT-proBNP.

	BNP	NT-proBNP	Clinical Implication	Reference(s)
Biochemical origin	Derived from proBNP cleavage; biologically active peptide	Inactive N-terminal fragment of proBNP released in equimolar amounts	Both reflect myocardial wall stretch and neurohormonal activation	[14,15,16]
Half-life	~20 min	~60–120 min	NT-proBNP remains elevated longer and better reflects chronic stress	[17]
Clearance pathway	Enzymatic degradation via neprilysin and natriuretic peptide receptors	Primarily renal clearance	NT-proBNP disproportionately elevated in renal impairment	[14,17]
Major confounders in ICU	Sepsis, mechanical ventilation, renal dysfunction, age, Neprilysin inhibitor	Renal dysfunction, age, inflammation	Interpretation must consider non-cardiac causes of elevation	[30,37]
Kinetic behavior during therapy	Declines rapidly with decongestion or reduced wall stress	Declines more slowly; reflects cumulative stress	BNP better for real-time response, NT-proBNP for overall severity	[38,42]
Diagnostic role	Rapid but less stable indicator of acute heart failure	More stable; useful for serial measurement and prognosis	Combined interpretation enhances diagnostic confidence	[29,34]

## Data Availability

The study data are available from the corresponding author upon reasonable request.
